# Supplementary material for: “Don’t Bring Me a Dog…I’ll Just Keep It”: Understanding Unplanned Dog Acquisitions Amongst a Sample of Dog Owners Attending Canine Health and Welfare Community Events in the United Kingdom
Source: Animals (Basel). 2021 Feb 25;11(3):605. doi: 10.3390/ani11030605 (PMC7996527; doi:10.3390/ani11030605)
Supplement: Supplementary file 1 [file animals-11-00605-s001.pdf]

Supplementary File 1: Interview schedule for semi-structured interviews about dog acquisition experiences

Section A – About your dog

- What is your dog's name?
- What is your dog's breed or type?
- Are they male or female?
- How old is he/she?
- How long have you had your dog?
- How old was your dog when you got him/her?
- Do you have any other dogs currently?
    - If yes, could you tell me a bit about that dog/those dogs?
- Have you had any dogs prior to this?
    - If yes, could you tell me a bit about that dog/those dogs?

Section B – Finding your dog

- Where did you get your dog from?
    - Why did you use this source?
    - How did you locate this source?
    - Did you look in any other places?
- How did you decide on your dog?
- When was the first time you met your dog?
- How many times did you meet your dog before you took them home?
    - Over what period of time?
- Who was involved in the process of looking for your dog?

Section C – Deciding to get a dog

- Who made the decision to get this dog?
- How long ago?
- Why did you want to have a dog?

Section D – Before finding your dog

- Did you already have a clear idea of what you wanted? For example, did you know what breed or type of dog you'd like, what size, etc.
    - If yes, why was this?
- Did you look for any information or ask anyone for advice before getting your dog?
    - Who did you ask/where did you look?
    - Approximately how long did you spend looking for information (was it a long or short process)?
- Did you change your mind or have any second thoughts during this process?
    - Was it an easy decision or did you re-think anything?

Section E – After acquiring your dog

- What, if anything, have you most enjoyed about owning your dog?
- Is there anything that's been different to what you expected?
- Would you recommend this breed or type of dog to potential owners?
    - Why/why not?
- Would you recommend the source you acquired your dog from to potential owners?
    - Why/why not?
- If you were looking for another dog, would you do anything differently?
    - Where would you look now?
- What advice would you give to family or friends who want to get a dog?
